# Supplementary material for: Fluorescent Silica Nanoparticles Targeting Mitochondria: Trafficking in Myeloid Cells and Application as Doxorubicin Delivery System in Breast Cancer Cells
Source: Int J Mol Sci. 2022 Mar 12;23(6):3069. doi: 10.3390/ijms23063069 (PMC8954043; doi:10.3390/ijms23063069)
Supplement: Supplementary file 1 [file ijms-23-03069-s001.zip › ijms-1609888-supplementary.pdf]

Supplementary Figures

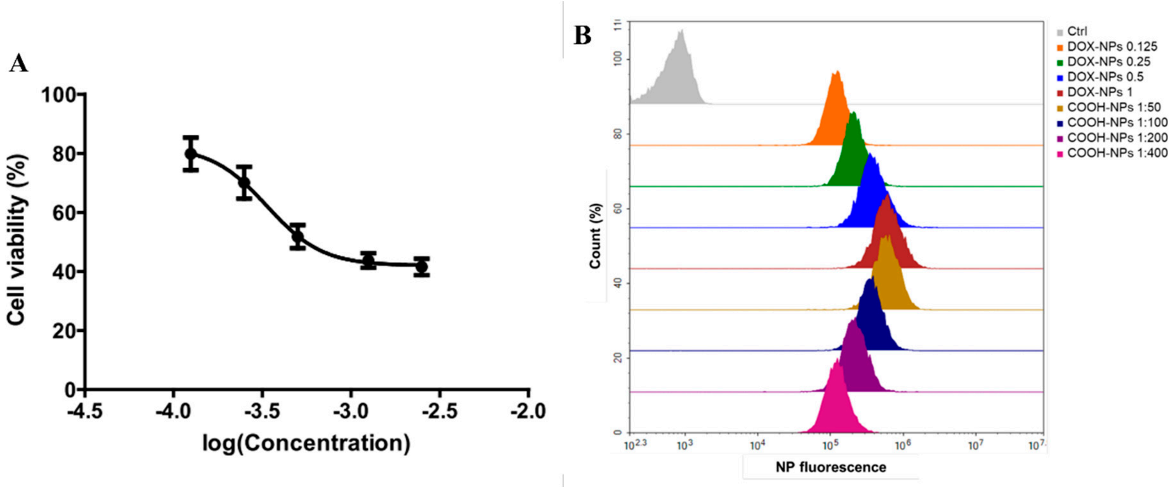

Figure S1. A The logarithmic curve for MCF-7 cells response to different concentration of doxorubicin at 24 h to calculate IC50 and use it in the further experiments. B Flow cytometry histogram overlay of MCF-7 cells incubated for 24 h with different concentrations of DOX-NPs (0.125, 0.25, 0.5 and 1 μg/mL of drug) and COOH-NPs (diluted 1:50, 1:100, 1:200 and 1:400), in order to titrate NPs and utilize stand-alone NP as control with the same fluorescence of DOX-NPs in the NP fluorescent channel.

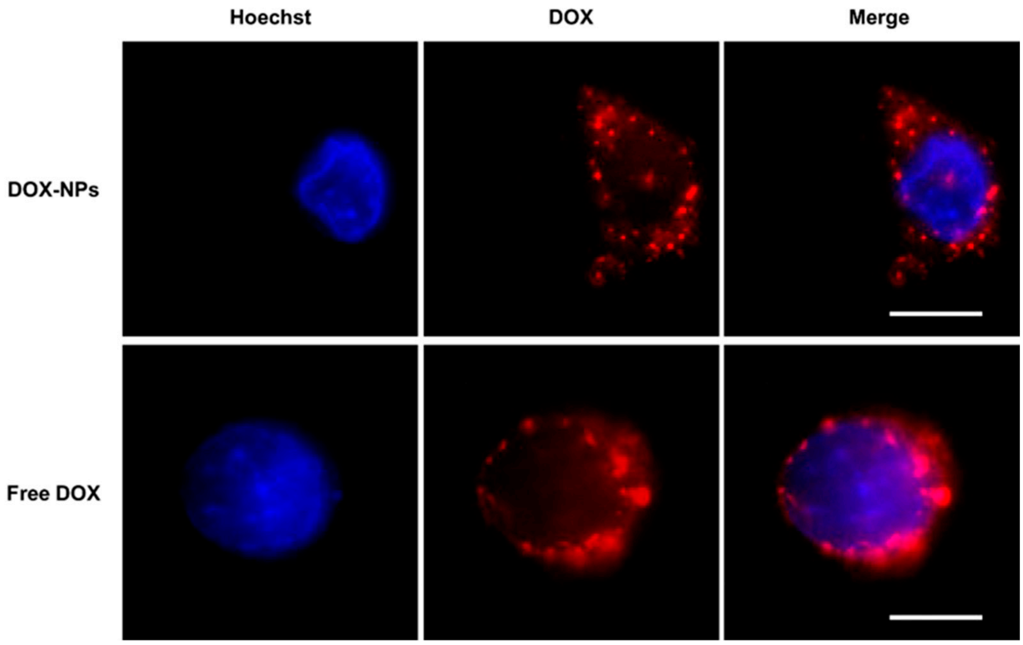

Figure S2. Fluorescent microscopy images of MCF-7 cells incubated for 48 h with DOX-NPs and free DOX and counter-stained nuclei with Hoechst 33342 to investigate doxorubicin localization inside the cells. Doxorubicin in red, Hoechst 33342 in blue. MCF-7 cells treated with DOX-NPs mainly present DOX fluorescent confined inside vesicle-like organelles (above frames), whereas free DOX is also inside the nucleus and in perinuclear region, besides micronuclei which are being disposed.

of by the dying cell (below frames). Merged images highlight the presence of free DOX into the nucleus, which results in a more violet color. Scale bar= 10 μm. Magnification 100X oil immersion.

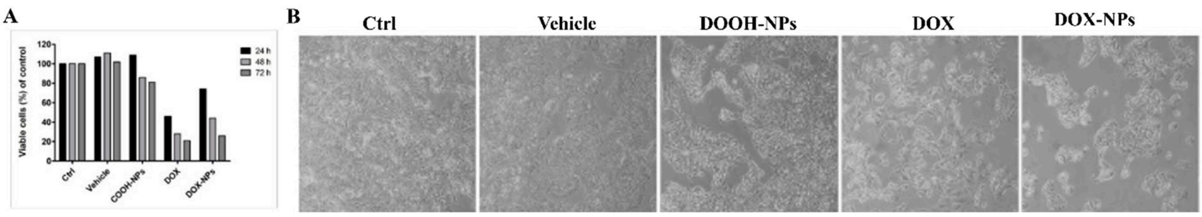

Figure S3. In vitro cytotoxicity and cell density of MCF-7 treated with COOH-NPs, DOX and DOX-NPs during time. MCF-7 were incubated with medium (Ctrl neg), DMSO (vehicle), COOH-NPs (diluted 1:50), free DOX (1 μg/mL) and DOX-NPs (1 μg/mL of DOX) for 24, 48 and 72 h at 37° C (n=2). (A) Cell viability was assessed through Trypan Blue exclusion assay and data are expressed as percentage of control. (B) Representative phase contrast images of MCF-7 with several treatments at 72 h to effect on morphology, cell density and detachment. Magnification 10X.

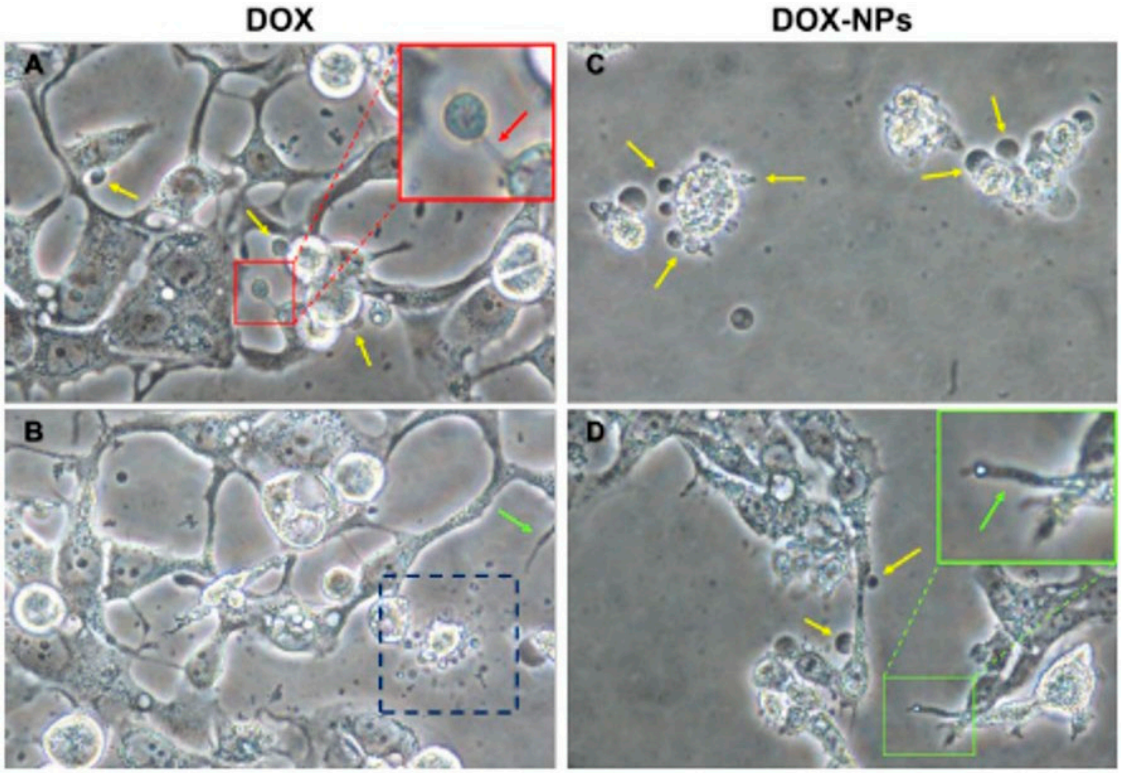

Figure S4. Some peculiar morphologic apoptotic features in MCF-7 cells treated with DOX and DOX- NPs. MCF-7 were incubated with free DOX (1 μg/mL) and DOX-NPs (1 μg/mL of DOX) for 24, 48 and 72 h at 37° C. Here we present some representative phase contrast images (magnification 40X) of MCF-7 treated for 72 h with DOX (A-B) and DOX.NPs (C-D), which present some characteristic aspects of the different stages of apoptotic until cell disassembly. Yellow arrows show apoptotic blebs on cell surface, in particular in frame C we observed two different stages: surface blebbing (on the left) involves the cell periphery while dynamic blebbing (on the right) occurs at a later stage and.

can lead to drastic changes in cell shape. After apoptotic membrane blebbing, a cell undergoes further morphological changes as generating a variety of membrane protrusions, with subtle differences in morphology, including microtubule spikes, apoptopodia and beaded apoptopodia, which are highlighted with green arrows. Green inset shows the presence of a beaded apoptodia. Lastly, the release of individual membrane-bound apoptotic bodies occurs and leads to the final step of cell fragmentation. The red inset underlines the release of an apoptotic body at the apex of a membrane protrusion, whereas the blue cage contains a cell which is going to fragmentation with the release of subcellular membrane-vesicles.

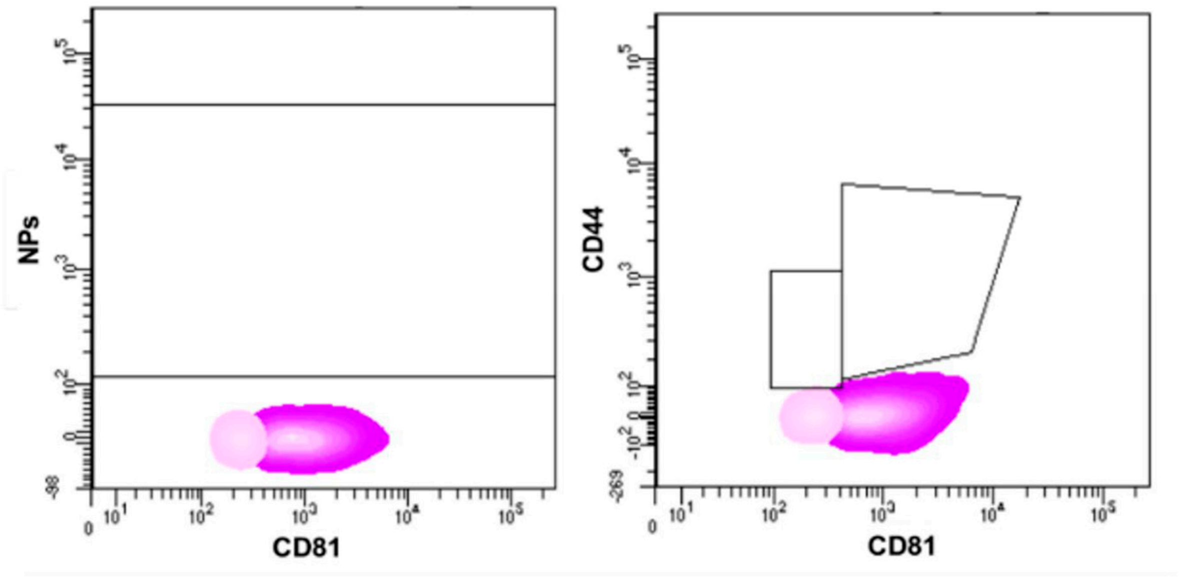

Figure S5. Flow cytometry contouring plots of negative sample (w/o CD44 RPE) to set the gates for the detection of NPs containing EVs (on the left), by distinguishing CD81- (pink) and CD81+ (violet) among CD44+ events (on the right) of supernatants. In order to count the events in the corresponding gate, media were stained with Dako CytoCountTM beads. At least 30000 events have been acquired by flow cytometry for each experimental condition.

Quantification of residual amine groups

Quantification of carboxylic groups on the surface was obtained using a colorimetric indirect method. COOH-NPs were made to react with fluorescein isothiocyanate (FITC, Abs max=494 nm, Em max= 518 nm) (Sigma-Aldrich, St Louis, MO, USA), which bound amine groups left on NP surface. 108 μL of COOH-NPs diluted 1:2 in buffer Carbonate/Bicarbonate 2X (pH=9) were mixed with FITC (10 mg/mL in DMSO, Molar Ratio=10) and incubated 1h at RT in the dark. The reaction mixture was applied on size exclusion chromatography column (Sephadex G25, GE Healthcare, Chicago, Il, USA) and eluted with buffer Carbonate/Bicarbonate 1X (pH=9). Selected fractions were collected and diluted with Carbonate/Bicarbonate to 3 mL of volume. The sample was then analysed at the

spectrophotometer (Agilent Technologies, Santa Clara, CA, USA), recording the whole spectrum. The absorption wavelength of FITC, used to calculate the moles of reacted FITC, directly proportional to -NH_2 left on NP surface, was found to be the FITC absorption maximum at the pH used for the reaction (pH=9.5; ε=80600 $M^{-1}cm^{-1}$; 1/ ε= 12.4 μM*cm).

The reaction yield was, therefore, 89 % of -COOH on SiNPs (Figure S6A).
The Figure S6B shows the absorption spectra of stand-alone SiNPs (dotted grey line) and DOX- NPs (solid blue line) in which are distinguishable both species, DOX, with maximum absorption at 500 nm, and SiNPs, with maximum absorption at 667 nm. Moreover, the SiNP peaks of the two spectra are almost superimposed, while the DOX one is well distinct.

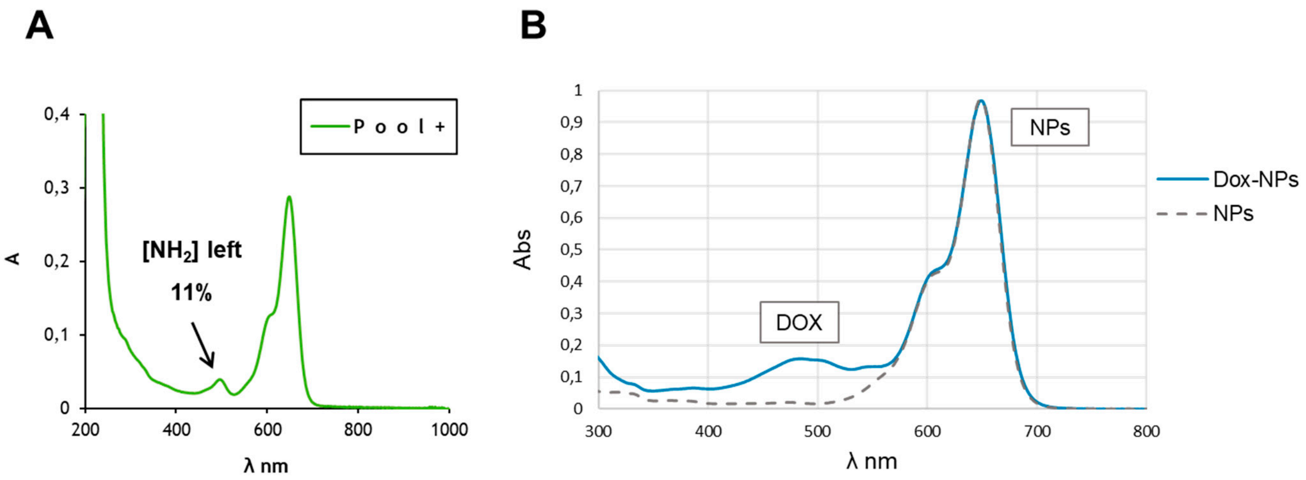

Figure S6. Characterization of DOX-NPs. (A) Quantification of residual amine groups on COOH-NPs surface. Absorption spectrum of COOH-NPs that were made to react with FITC. The peak highlighted by black arrow refers to the 11% of amine groups left on NP surface. (B) Absorption spectra of DOX conjugated (solid blue line) and stand-alone (dotted grey line) nanoparticles.

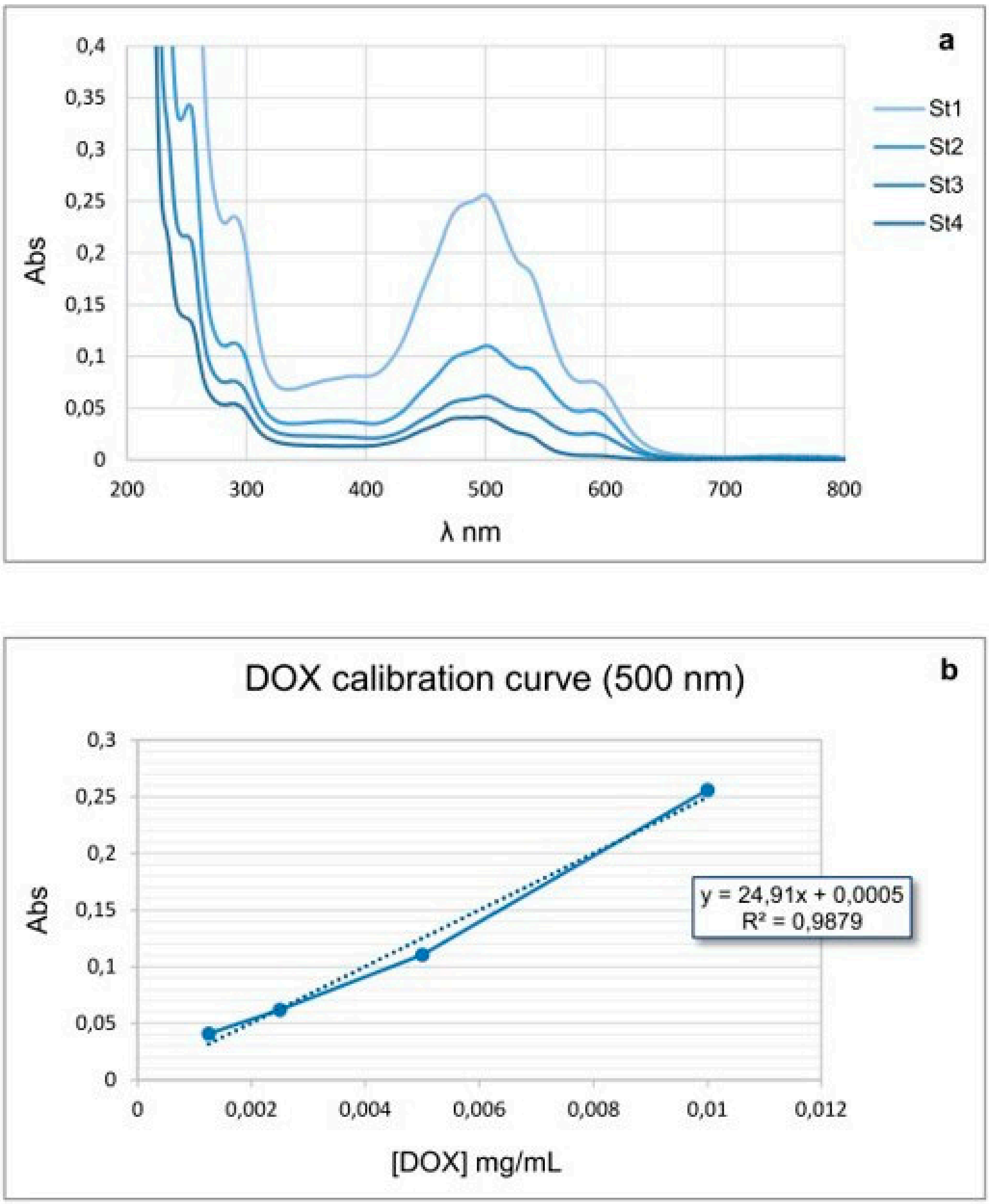

Figure S7. Doxorubicin spectrofluorimetric calibration curve for drug quantification. (a) Absorption spectra of standard dilutions of DOX in ddH2O. (b) Calibration curve showing DOX concentration related to absorbance at 500 nm.

DOX-NPs	
Fluorophore	Cy5
[Dye]	18.87 µM
ε ***($M^{-1}cm^{-1}$)***	102600
λ max Abs (nm)	648 nm
λ max Em (nm)	667 nm
[NH_2] left	11%
COOH surface content	243.78 µM
[DOX]	5.2 µg/mL

Table S1 Summary of physicochemical features of DOX-NPs.
